# Diagnostic Accuracy of Lipopolysaccharide-Binding Protein as Biomarker for Sepsis in Adult Patients: A Systematic Review and Meta-Analysis

**DOI:** 10.1371/journal.pone.0153188

**Published:** 2016-04-07

**Authors:** Kuan-Fu Chen, Chung-Hsien Chaou, Jing-Yi Jiang, Hsueh-Wen Yu, Yu-Hsiang Meng, Wei-Chen Tang, Chin-Chieh Wu

**Affiliations:** 1 Department of Emergency Medicine, Chang Gung Memorial Hospital, Keelung, Taiwan; 2 Clinical Informatics and Medical Statistics Research Center, Chang Gung University, Taoyuan, Taiwan; 3 Community Medicine Research Center, Chang Gung Memorial Hospital, Keelung, Taiwan; 4 Department of Emergency Medicine, Chang Gung Memorial Hospital, Linkou, Taiwan; 5 School of Traditional Chinese Medicine, Chang Gung University, Taoyuan, Taiwan; 6 School of Medicine, Chang Gung University, Taoyuan, Taiwan; McGill University, CANADA

## Abstract

**Introduction:**

Lipopolysaccharide-binding protein (LBP) is widely reported as a biomarker to differentiate infected from non-infected patients. The diagnostic use of LBP for sepsis remains a matter of debate. We aimed to perform a systematic review and meta-analysis to assess the diagnostic accuracy of serum LBP for sepsis in adult patients.

**Methods:**

We performed a systematic review and meta-analysis to assess the accuracy of LBP for sepsis diagnosis. A systematic search in PubMed and EMBASE for studies that evaluated the diagnostic role of LBP for sepsis through December 2015 was conducted. We searched these databases for original, English language, research articles that studied the diagnostic accuracy between septic and non-septic adult patients. Sensitivity, specificity, and other measures of accuracy, such as diagnostic odds ratio (DOR) and area under the receiver operating characteristic curve (AUC) of LBP were pooled using the Hierarchical Summary Receiver Operating Characteristic (HSROC) method.

**Results:**

Our search returned 53 reports, of which 8 fulfilled the inclusion criteria, accounting for 1684 patients. The pooled sensitivity and specificity of LBP for diagnosis of sepsis by the HSROC method were 0.64 (95% CI: 0.56–0.72) and 0.63 (95% CI: 0.53–0.73), respectively. The value of the DOR was 3.0 (95% CI: 2.0–4.0) and the AUC was 0.68 (95% CI: 0.64–0.72). Meta-regression analysis revealed that cut-off values accounted for the heterogeneity of sensitivity and sample size (> = 150) accounted for the heterogeneity of specificity.

**Conclusions:**

Based on the results of our meta-analysis, LBP had weak sensitivity and specificity in the detection of sepsis. LBP may not be practically recommended for clinical utilization as a single biomarker.

## Introduction

Despite the recent focus on the importance of early and effective therapy for sepsis in past decades, this fatal disease still poses a burden to critically ill populations worldwide [1]. One of the main reasons first-line health care providers fail to provide timely and accurate care to patients with sepsis is the lack of rapid and accurate diagnostic aids. During the past decade, even with the abundant progress made in the field of molecular biology, clinicians still rely on microbiological culture as the conventional reference standard to distinguish sepsis from non-infectious conditions. These cultures can take days to receive results, and lack sensitivity. Thus, there is an urgent need for a rapid, simple, and accurate method to enhance sepsis diagnosis.

Lipopolysaccharide (LPS)-binding protein (LBP) was initially discovered as an acute-phase reactant binding with the LPS of Gram-negative bacteria walls to form LPS-LBP complexes [2, 3] and was later found to be elevated in gram-positive bacteremia [4]. The LPS-LBP complex binds to CD14 and to the Toll-like receptor 4/MD2-complex and results in transcription of cytokines and other pro-inflammatory mediators [5, 6]. Interestingly, LBP is constitutively present at a concentration of 5 to 10 μg/ml in healthy human serum [7].

During sepsis, LBP levels were found to increase to median peak levels of 30–40 μg/ml within 24 h, almost seven times higher than normal levels [7–9]. These properties made LBP a promising tool for the diagnosis of sepsis, and suitable to discriminate between non-septic systemic inflammatory response syndrome (SIRS) and sepsis [10]. However, further studies indicate that LBP is a rather non-specific marker for inflammatory response [7, 9]. Whether LBP is appropriate for use as a clinical diagnostic tool for the differentiation between infectious and noninfectious etiologies of SIRS is still uncertain [9].

Recently, several studies have investigated the role of serum LBP in differentiating sepsis from non-infectious SIRS in different clinical settings [7, 9, 11–16]. However, due to the limited sample size recruited in these studies, the results are not consistent. Therefore, we aimed to perform a systematic review and meta-analysis to assess the diagnostic accuracy of serum LBP for sepsis in adult patients.

## Methods and Materials

### Literature Search Strategy

A protocol was developed prior to the conduction of this systematic review and meta-analysis. We conducted a comprehensive systematic search in PubMed and EMBASE for studies that evaluated the diagnostic accuracy of LBP for sepsis through December 2015. The following keywords were used: ((“Systemic Inflammatory Response Syndrome” OR “SIRS”) AND “Sepsis”) AND (“Early Diagnosis” OR “Diagnosis”) AND “Lipopolysaccharide-binding protein” AND “adult” in PubMed and (sensitivity OR diagnostic AND accuracy:lnk OR diagnostic AND ('sepsis'/exp OR sepsis) AND (' LBP' OR LBP) AND [english]/lim) in EMBASE. We searched these databases for original, English language, research articles that studied the diagnostic accuracy between septic and non-septic in adult patients. Further data was uncovered through review of the reference lists from the primary articles, and Google searches.

### Study Selection

After the removal of duplicate references, two reviewers (JYJ, and HWY) independently screened, and decided the inclusion of studies in this review using the following inclusion criteria: (1) sepsis related biomarker studies; (2) diagnostic instead of prognostic studies: e.g. studies on the diagnosis of sepsis instead of those on mortality prediction; and (3) articles in English. Studies were independently excluded based on the following exclusion criteria: (1) non-sepsis biomarker related study; (2) non-diagnostic study; (3) non-original study: e.g. literature review, editorial piece; (4) no performance parameters given (i.e. sensitivity, specificity, and 2x2 contingency tables). The Cohen’s Kappa statistic [17] was evaluated for the agreement with study inclusion.

### Data Extraction

Two reviewers (JYJ, and HWY) independently extracted all relevant information from each study such as study setting, material and method, statistical method, and the results via electrical form (Microsoft Access). We abstracted the numbers of true-positive, false-positive, false-negative, and true-negative based on the provided indices of sensitivity, specificity, and sample size values.

### Quality Assessment

The Quality Assessment of Diagnostic Accuracy Studies (QUADAS-2) checklist [18] was used to assess the methodological quality of included studies. QUADAS-2 is a four-domain tool, including patient selection, index test, reference standard, flow and timing, to assists authors of systematic reviews in rating the risk of bias and the applicability of diagnostic accuracy from their studies. Each domain is assessed in terms of risk of bias, and the first three domains are also assessed regarding applicability. Signaling questions are included to help judge risk of bias. For both categories, risk of bias and applicability, and the individual criteria was classified as low risk, high risk, or unclear. If and only if the answers to all questions of a domain were judged as “yes” indicating low risk of bias, then this domain was judged to be at “low” risk of bias. If any question of a domain was judged as “unclear,” then the domain would be judged as “unclear”. Two of the authors (CCW and WCT) assessed risk of bias independently, and discrepant results were resolved in a consensus meeting. Agreement between the two reviewers for assessment of methodological quality was evaluated using the Cohen’s Kappa statistic [17]. Subgroup analysis was conducted subsequently according to the results of the methodological quality assessment. Consensus meetings solved all discrepant results.

### Data synthesis and Statistical Analysis

We calculated the pooled sensitivity and specificity, diagnostic odds ratio (DOR) and the area under the receiver operating characteristic curve (AUC) based on the Hierarchical Summary Receiver Operating Characteristic (HSROC) method for meta-analysis of diagnostic test data [19]. It is believed that this approach could maximize use of the available data from each study, irrespective of the threshold used. We also constructed the respective summary receiver operating characteristic curves (SROC) [20] and calculated the AUC, irrespective of the different cut-off points used. For heterogeneity, it can be caused by two effects, threshold and non-threshold. For threshold effect, it was the calculation of spearman correlation coefficient (ρ) between logarithms of sensitivity and (1-specificity). Furthermore, for non-threshold effect, it was quantified by applying the chi-square (χ^2^) and the Cochrane-Q test (for DOR) and by determining the *I*^2^ metric [21]. Owing to the high statistical heterogeneity found among these studies, we performed univariable meta-regression analyses using a bivariate model

$$logit\left(TPR_{i}\right)=\beta_{01}+\beta_{11}\left(covariate\right)$$

$$logit\left(FPR_{i}\right)=\beta_{02}+\beta_{12}\left(covariate\right)$$

to find the source of variability by potential factors. Meta-regression is a sophisticated tool for exploring heterogeneity. It aims to discern whether a linear relationship exists between an outcome measure and one or more covariates. The associations found in a meta-regression should be considered hypothesis generating and not regarded as proof of causality. We subsequently conducted subgroup analyses for studies with adequate number (more than three required for HSROC). Given the possibility of a small number of included studies, we also planned to perform subgroup analyses for covariates that were deemed to be clinically significant as predictors of accuracy, in order to avoid the low statistical power of the meta-regression. To test for possible publication bias, we constructed Deeks’ effective sample size funnel plots versus the diagnostic odds ratio and did a regression test of asymmetry. All statistical tests were two-sided and statistical significance was defined as *p*-value<0.05. The Midas module for Stata 13.1 (Stata Corporation, College Station, TX, USA) was used for all statistical and meta-analyses. We used mada package in R (version 3.1.3) to complete the bivariate binomial mixed-effect meta-regression model. Midas and the QUADAS modules for Stata were used for all graphical displays of the quality of the included studies.

## Results

### Identification of studies

**Fig 1:**
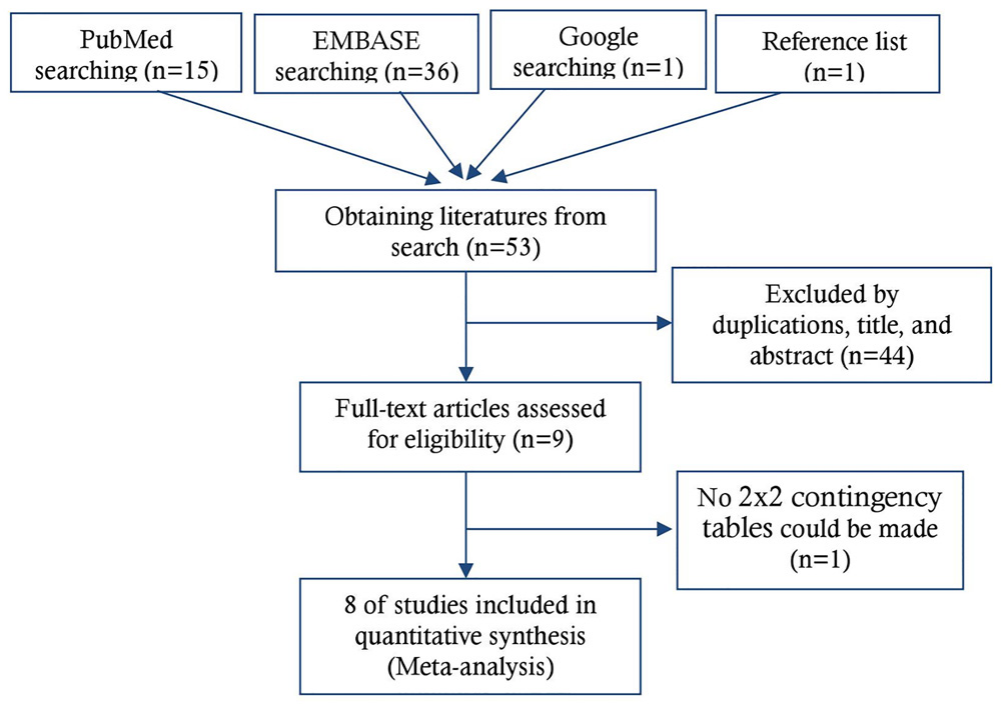
Study identification, inclusion, and exclusion for meta-analysis.

Overall, our electronic search yielded 53 published studies, among them, one from the Google searching and another from the reference lists. After excluding 44 studies irrelevant to this review by screening titles and abstracts (six duplicates, 19 non-sepsis biomarker related studies, 13 non-diagnostic studies, one non-original editorial piece, three studies without performance parameters given, and two confidence proceedings), nine studies with available full-text were included for further data extraction procedures. Having reviewed the full text of the remaining nine articles, we then excluded another study for lack of information available to make a 2x2 contingency table for comparisons. Finally, eight eligibility studies were included in the analysis [7, 9, 11–16] (Fig 1). The Cohen’s Kappa statistic evaluated agreement on study inclusion was 0.94 (95% CI: 0.83–1.00), indicating almost perfect inter-rater agreement.

### Study characteristics

The main characteristics of included studies are summarized in Table 1. Of the 1684 patients included in our study, 486 (29%) were hospitalized in ICUs, 746 (44%) were admitted in the EDs, 452 (27%) were admitted in the general wards, 506 (30%) had sepsis, 308 (18.29%) had SIRS, and 1140 (51.71%) were normal healthy controls. All studies were conducted prospectively, and all studies used a case-control design. Regarding the reference tests, seven studies [7, 9, 11–13, 15, 16] defined the ‘gold standard’ by the criteria defined in the 1992 ACCP/SCCM consensus conference [22], and one [14] by blood culture. The proportion of patients with sepsis among studies ranged widely from 16% to 87% (mean 33%, Table 1). The year of study conducted ranged from 2003 to 2013. For the types of specimen tested for LBP detection, five [7, 9, 11, 14, 16] were serum, three [12, 13, 15] were plasma. All studies used the chemiluminescent LBP assay.

**Table 1:** Main Characteristics of Selected Studies.

Author, year	Study design	Mean age (years) Total or Case/ Control	Patient source	Case/ Control (n)	Total (n)	Proportion of patients with sepsis (%)	Specimen tested	Cut-off (μg/ml)	Sensitivity, specificity	TP(n) (%)	FP(n) (%)	FN(n) (%)	TN(n) (%)
**Gaini, S., et al., 2007 [**15**]**	Prospective	56.8/56.9	General Ward	134/20	154	87	Plasma	64.6	0.79, 0.5	106 (68.8)	10 (6.5)	28 (18.2)	10 (6.5)
**Gille-Johnson, P., et al., 2012 [**14**]**	Prospective	61	ED	118/286	404	29	Serum LBP	36	0.76, 0.45	90 (22.3)	157 (38.9)	28 (6.9)	129 (31.9)
**Meynaar, I.A., et al., 2011 [**16**]**	Prospective	68/65	ICU	32/44	76	42	Serum LBP	30	0.53, 0.91	17 (22.4)	4 (5.3)	15 (19.7)	40 (52.6)
**Nierhaus, A., et al., 2013 [**11**]**	Prospective	52.9	ICU	51/19	70	73	Serum LBP	Not provided	0.67, 0.64	34 (48.6)	8 (11.4)	17 (24.3)	11 (15.7)
**Prucha, M., et al., 2003 [**9**]**	Prospective	45.2/49.7	ICU	28/40	68	41	Serum LBP	29.8	0.50, 0.74	14 (20.6)	10 (14.7)	14 (20.6)	30 (44.1)
**Ratzinger, F., et al., 2013 [**12**]**	Prospective	58/60	General Ward	75/223	298	25	Plasma	18.2	0.58, 0.67	43 (14.4)	73 (24.5)	32 (10.7)	150 (50.4)
**Sakr, Y., et al., 2008 [**7**]**	Prospective	63	ICU	64/208	272	23	Serum LBP	32	0.60, 0.62	38 (14.0)	79 (29.0)	26 (9.6)	129 (47.4)
**Tromp, M., et al., 2012 [**13**]**	Prospective	59/59	ED	55/287	342	16	Plasma	27.3	0.60, 0.68	33 (9.6)	92 (26.9)	22 (6.4)	195 (57.1)

*Note*. TP = True positive; FP = False positive; FN = False negative; TN = True negative

### Results of Quality assessment

**Fig 2:**
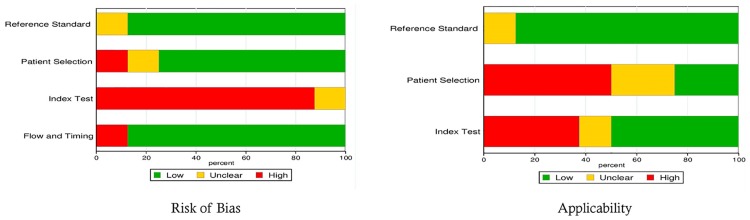
Graphical Display of 8 studies results (QUADAS-2).

Table 2 and Fig 2 summarized the methodological quality assessment with the QUADAS-2 tool of the eight included studies. For the risk of bias in the reference standard, seven studies scored “low” since they used the criteria defined in the ACCP/SCCM consensus conference [22] as the gold standard. Regarding the domain of risk of bias in patient selection, six studies that provided a clear definition of the exclusion criteria and one study that only described the basic information about subjects without exclusion criteria were scored “low” and “high” risk, respectively. One study that did not show enough information regarding how they excluded patients was scored “unclear”. Regarding the domain of index test, it contains two terms, “Were the index test results interpreted without knowledge of the results of the reference standard?”, and “If a threshold was used, was it pre-specified”. If and only if the answers to these two terms were judged as “yes” indicating low risk of bias, otherwise, it was judged as high risk. In our included studies, seven studies did not pre-specify the threshold, so we decided to judge these studies to have “high” risk of bias regarding “Index test”. For patient flow and timing domains, seven studies scored “low” since they clearly defined the appropriate interval between the index test and reference standard in their studies. In relation to applicability, the reference standard domain scored well for seven of the eight included studies. Patient selection criteria in two studies were in accordance with our analysis inclusion criteria and scored“low”. The Cohens Kappa statistic for inter-rater agreement of assessment of quality was 0.37 (95% CI: 0.24–0.52). However, all disagreed evaluations were resolved after the consensus meeting.

**Table 2:** Quality Assessment for 8 studies (QUADAS-2).

Study	Risk of bias				Applicability concerns		
	Patient selection	Index test	Reference standard	Flow and timing	Patient selection	Index test	Reference standard
Gaini, S., et al., 2007 [15]	☺	☹	☺	☺	☹	☺	☺
Gille-Johnson, P., et al., 2012 [14]	☺	☹	?	☺	?	?	?
Meynaar, I.A., et al., 2011 [16]	☺	☹	☺	☺	☹	☺	☺
Nierhaus, A., et al., 2013 [11]	☺	?	☺	☺	☹	☹	☺
Prucha, M., et al., 2003 [9]	?	☹	☺	☺	☺	☺	☺
Ratzinger, F., et al., 2013 [12]	☹	☹	☺	☺	?	☹	☺
Sakr, Y., et al., 2008 [7]	☺	☹	☺	☺	☹	☺	☺
Tromp, M., et al., 2012 [13]	☺	☹	☺	☹	☺	☹	☺

☺ Low Risk ☹ High Risk ? Unclear Risk

### Results of quantitative data synthesis

**Fig 3:**
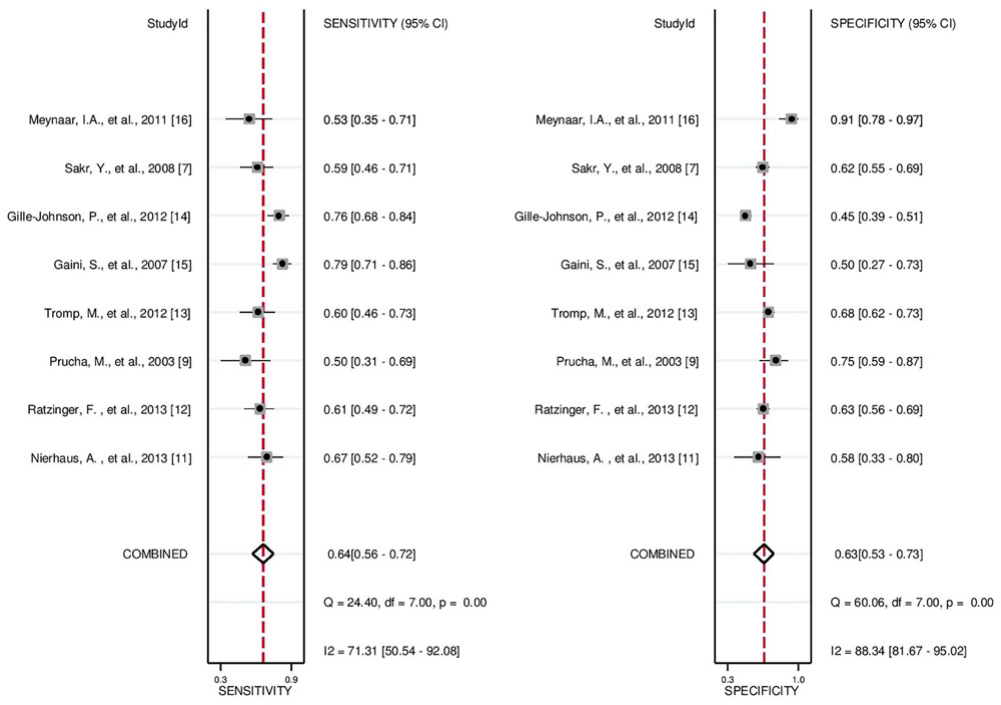
Forest plots of the sensitivity and specificity for serum LBP level across all included studies.

**Fig 4:**
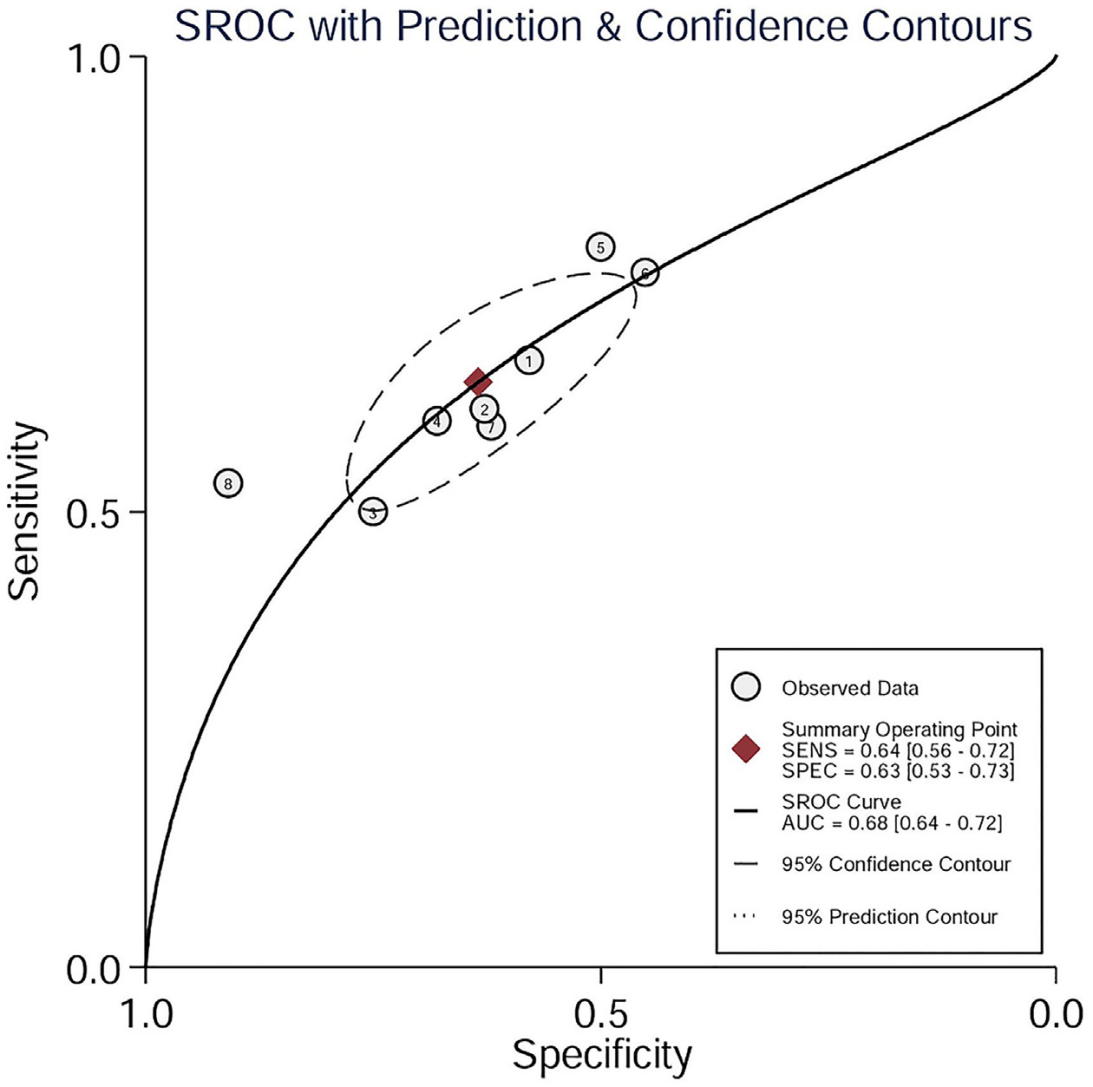
Hierarchical summary receiver operating characteristic plot of serum LBP level across all included studies. 1: Axel Nierhaus 2013 [11]; 2: Y.Sakr 2008 [7]; 3: M.Prucha 2003 [9]; 4: M.Tromp 2012 [13]; 5: S.Gaini 2007 [15]; 6: P.Gille-Johnson 2012 [14]; 7: Franz Ratzingerl 2013 [12]; 8: Iwan A.Meynaar 2011 [16]

The sensitivity reported by the eight studies ranged from 0.50–0.79, and the specificity ranged from 0.45–0.91. Pooled sensitivity and specificity estimates of all included studies obtained by the HSROC methods were 0.64 (95% CI: 0.56–0.72) and 0.63 (95% CI: 0.53–0.73), respectively (Figs 3 and 4). We also constructed summary ROC for LBP, and the result showed the AUC of LBP to be 0.68 (95% CI: 0.64–0.72, Fig 4). The diagnostic odds ratio (DOR) of the included studies ranged from 2.39 (95% CI: 1.35–4.23) to 11.33 (95% CI: 3.28–39.18), and the pooled DOR was 3.0 (95% CI: 2.0–4.0), indicating a wide range and a poor level of overall accuracy. Our analysis indicated that serum LBP has a poor degree of diagnostic accuracy for sepsis.

### Investigation of heterogeneity test

From visual inspection of the SROC curve (Fig 4) and the estimation of the Spearman correlation coefficient (ρ = -1.00, *p*-value <0.001), we could conclude that threshold effect existed and contributed to the heterogeneity in our study. On the other hand, for the non-threshold effect, the heterogeneity was measured in the overall sensitivity (χ^2^ = 71.31, *p*-value<0.001, *I*^2^ = 71.31), specificity (χ^2^ = 60.06, *p*-value<0.001, *I*^2^ = 88.34), and DOR (Cochrane-Q = 21.81, *p*-value<0.001, *I*^2^ = 44.07). Therefore, it indicated that threshold and non-threshold effects accounted for the high heterogeneity. We subsequently performed the bivariate meta-regression analysis to explore the source of heterogeneity. Mean age, patient source, patient sample size, proportion of patients with sepsis, types of specimen, and cut-off value were used as covariates. The results of bivariate meta-regression analysis (Table 3) revealed that cut-off value accounted for the heterogeneity of sensitivity and sample size (> = 150) accounted for the heterogeneity of specificity.

**Table 3:** The results of univariable meta-regressions.

	Sensitivity			Specificity		
Covariates	Coefficient	SE	*p*-value	Coefficient	SE	*p*-value
**Mean age**	-0.001	0.034	0.968	-0.007	0.040	0.854
**Patient source (ICU vs non-ICU)**	0.570	0.294	0.053	0.616	0.348	0.077
**Sample size (> = 150)**	0.472	0.336	0.160	0.866	0.382	0.023
**Proportion of patients with sepsis (>0.5)**	0.623	0.341	0.068	0.488	0.495	0.325
**Specimen**	-0.276	0.350	0.430	-0.213	0.395	0.590
**Cut-off**	0.021	0.009	0.029	0.015	0.015	0.315

### Results of subgroup analysis

In subgroup analysis, the results of patient sample size revealed that in the three studies [9, 11, 16] with smaller sample sizes (sample size<150), the pooled sensitivity and specificity were 0.58 (95% CI: 0.45–0.69) and 0.77 (95% CI: 0.53–0.91), respectively, and in the other five studies [7, 12–15] with larger sample sizes (sample size>150), the pooled sensitivity and specificity were 0.68 (95% CI: 0.58–0.77) and 0.57 (95% CI: 0.53–0.66), respectively.

We summarized the LBP cut-off values utilized in those studies in Table 1. Cut-off values for serum LBP varied between studies, ranging from 18.2 to 64.6 μg/ml. We calculated the pooled sensitivity and specificity for these studies [7, 9, 13, 14, 16] with similar cut-off values (ranged from 27.3 to 36 μg/ml). The pooled sensitivity and specificity were 0.61 (95% CI: 0.49–0.71) and 0.68 (95% CI: 0.53–0.80) for these five studies, respectively.

In relation to proportion of patients with sepsis, it demonstrated that in the six studies [7, 9, 12–14, 16] with relatively lower proportion (<50%), the pooled sensitivity and specificity were 0.61 (95% CI: 0.51–0.69) and 0.67 (95% CI: 0.55–0.77), respectively. Additionally, in relation to the reference standard, it demonstrated that in the seven studies [7, 9, 11–13, 15, 16] with the same gold standard (defined by the criteria defined in ACCP/SCCM consensus conference), the pooled sensitivity and specificity were 0.62 (95% CI: 0.53–0.70) and 0.65 (95% CI: 0.56–0.74), respectively.

Furthermore, among the included studies, four [7, 9, 11, 16] were conducted in the ICU, two [13, 14] in the emergency department, and two [12, 15] in the general ward. The pooled sensitivity and specificity were 0.58 (95% CI: 0.49–0.66) and 0.72 (95% CI: 0.54–0.85), respectively, in ICU patients, indicating a slightly better specificity. However, for the other four “non-critical” patients, the pooled sensitivity and specificity were 0.70 (95% CI: 0.59–0.79) and 0.56 (95% CI: 0.43–0.68), respectively, indicating a slightly better sensitivity.

We also performed subgroup analyses for the different types of specimen used to detect LBP concentrations. The pooled sensitivity and specificity were 0.62 (95% CI: 0.51–0.72) and 0.66 (95% CI: 0.50–0.80), respectively, for serum specimen. Otherwise, for plasma specimen, the pooled sensitivity and specificity were 0.66 (95% CI: 0.5–0.8) and 0.64 (95% CI: 0.54–0.72), respectively. The confidence intervals of the pooled sensitivity and specificity are seems completely overlapping for serum and plasma specimens.

### Results of publication bias

**Fig 5:**
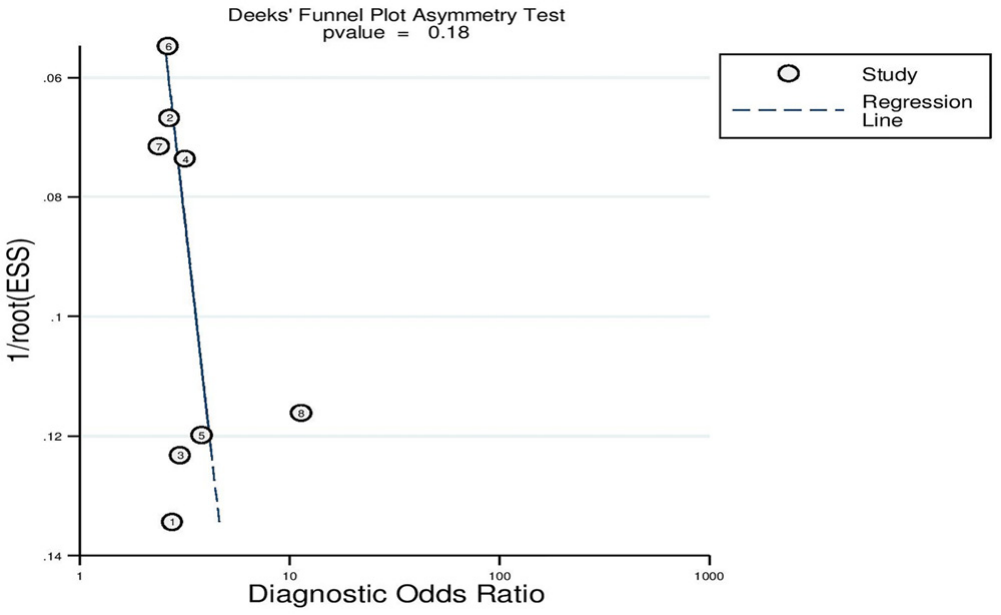
Deeks’ funnel plot asymmetry test for publication bias.

Fig 5 displays the Deeks’ effective sample size funnel plot and the regression test of asymmetry of the included studies. The Deeks’ test was not statistically significant (*p*-value = 0.18) indicating that there is no direct evidence for publication bias.

## Discussion

It is critical to identify sepsis early in the clinical course to provide timely and accurate treatment for patients. Clinical assessment or non-specific criteria such as SIRS alone cannot satisfy this purpose. Diagnosis of sepsis is still largely based on SIRS in the presence of an infectious focus. SIRS, purposed since the consensus meeting in 1992, was originally developed for research purposes, and is neither sensitive nor specific in diagnosing sepsis. Furthermore, other than sepsis, SIRS can be found in various non-septic conditions, including trauma, surgery, and pancreatitis [23, 24]. There is consensus regarding the need to develop an ideal marker of sepsis, which can be measured rapidly and easily, sensitive enough to detect infection in patients, and specific enough to rule out non-infectious SIRS [25].

A growing number of studies suggest that LBP may play a role in identifying sepsis, and our study summarizes the overall diagnostic performance of LBP for sepsis based on the current available literature. To our knowledge, this is the first systematic review and meta-analysis to summary this biomarker, Lipopolysaccharide- Binding Protein (LBP), in diagnosis of adult patients with sepsis. Our results reveal that the pooled sensitivity of serum LBP for the diagnosis of sepsis to be sub-optimal (pooled sensitivity 0.64 and pooled specificity 0.63). The AUC used to summarize the overall diagnostic performance of the included studies was not satisfactory (0.68). In this meta-analysis, our pooled analysis indicated that serum LBP seemed to have a moderate to low diagnostic accuracy for sepsis.

The DOR combines the strengths of sensitivity and specificity, and has the advantage of accuracy as a single indicator, with higher values indicating the highest accuracy. In this meta-analysis, the DOR of the included studies ranged from 2.39 (95% CI: 1.35–4.23) to 11.33 (95% CI: 3.28–39.18), and the pooled DOR was 3.0 (95% CI: 2.0–4.0), indicating a wide range and a low level of overall accuracy. In Wu et al. [26], they suggest that the disparity between these included studies may result from the patient sample size variations. Song F. et al. [27] was suggested that small studies tend to overestimate the DOR and studies based on small sample sizes may have allowed higher type II errors.

There are several other sources that may cause the heterogeneity of the included studies surveying the performance of LBP in our study. The specificity of LBP seemed to be slightly better in the ICU setting (0.72), low sepsis prevalence (0.67), and smaller sample size (0.71) from our results of subgroup analyses. Furthermore, the pooled sensitivity of LBP seemed to be slightly better in the non-critical setting (0.70). There are studies [7–9] supporting LBP as an early biomarker for sepsis, which could be the reason why LBP has the better specificity in ICU setting.

Several biomarkers including procalcitonin (PCT) and C-reactive protein (CRP) are already available for clinical use to diagnose sepsis; however, their effectiveness in distinguishing sepsis from other inflammatory or for predicting outcome is limited by their far from perfect specificity and sensitivity. A clinical study performed by Meynaar et al. [16] showed that PCT was more useful (the area under the ROC curve (0.95, 95% CI: 0.90–0.99)) than LBP, CRP, and IL-6 in differentiating sepsis from SIRS in critically ill patients admitted to hospital. However, a recent meta-analysis published by Wacker et al. [28], concluded that the accuracy of PCT to discriminate sepsis and SIRS was sub-optimal as well, and the sensitivity and specificity of PCT in diagnosing sepsis were 0.77 (95% CI 0.72–0.81) and 0.79 (95% CI 0.74–0.84), respectively. Due to the complexity of the sepsis response, current methods and biomarkers used for the diagnosis of sepsis remain unsatisfactory [29], and more reliable diagnostic markers are needed.

Recently, many biomarkers have been proposed for use in sepsis diagnosis. However, none has sufficient sensitivity or specificity to be the single test able to distinguish sepsis from other inflammatory conditions. In view of the complexity of the sepsis response, it is unlikely that a single ideal biomarker will ever be found to diagnose sepsis [30]. From the results of Gibot et al. [31], they suggested that a combination of several biomarkers, such as the PMN CD64 index, PCT, and sTREM-1, may be more effective to improve diagnostic accuracy for sepsis. From the view of their suggestion, we may combine the measurement of LBP with other more sensitive biomarkers to increase the diagnostic accuracy for sepsis although LBP has a weak important role in diagnosing sepsis from our meta-analysis.

This systematic review and meta-analysis had several limitations that should be discussed. First, the number of included studies for LBP was still small, although an extensive literature search was conducted. We included only eight studies published because of our strict inclusion criteria. Owing to the clinical and statistical heterogeneity, our study may have lacked statistical power to draw a definite conclusion, and more clinical diagnostic studies are needed to reach a final conclusion. Second, there are risks of bias regarding patient misclassification, since the reference standard utilized in these included studies varied in the patients who were clinically diagnosed as having sepsis without microbiological evidence. Third, we could not determine the ideal cut-off point for serum LBP test in the meta-analysis, since we did not have the raw data to map out the ROC curve. To determine whether there is a single threshold or a few important thresholds, further studies should be attempted to obtain all the original data. Finally, we only included studies published in English, which may have resulted in language bias, and the inclusion of other language publication or studies with null results may have yielded different results.

## Conclusions

Our systematic review summarizes evidence regarding the diagnostic accuracy of LBP as a biomarker for sepsis in adult patients. Based on the results of our meta-analysis, LBP had weak sensitivity and specificity. LBP may not be practically recommended for clinical utilization as a single biomarker.

## Supporting Information

S1 PRISMA Checklist Prisma checklist. (DOCX)
